# A novel encryption protocol for facilitating de-identification of genomics health data

**DOI:** 10.23889/ijpds.v9i5.2907

**Published:** 2024-09-18

**Authors:** David Copeland, Alasdair Taylor

**Affiliations:** 1 Datavant

The exponential growth and affordability of genomics sequencing in the clinical landscape in recent years have intensified a challenge facing privacy experts. They grapple with how to evaluate and mitigate the significant risk of genomics data being used to re-identify individuals. The lack of clear genomics guidance in HIPAA has invited a wide spectrum of expert opinion from advocating for conservative limitations to be placed on genomics data sharing to ‘security’-based arguments that carefully calibrated technical, administrative, and legal safeguards may provide adequate protection in many cases. The need to weigh privacy concerns against the extraordinary value that genomics data offers to cancer and rare disease clinical research is compounded by the reality that conventional data modifications, such as redaction, aggregation, and perturbation, often critically undermine these important use cases.

We present an innovative solution for de-identifying genomics data according to a conservative interpretation of HIPAA while still preserving maximal utility for clinical purposes. Our solution rejects the notion that privacy can be achieved directly via security. Instead, we introduce a reversible encryption protocol designed to be applied to specific combinations of genetic variants and their locations on the genome. The encrypted genomic data is fully de-identified, allowing researchers to perform analyses on the encrypted data in a secure Clean Room environment. The environment host is then able to decrypt and deliver variant-level summary statistics and aggregated outputs from the analysis to the recipient. As information in this format no longer links to patient-level data the solution remains de-identified end-to-end.

